# Arbuscular mycorrhizal fungi mediated biotic carbon pump in alpine meadows of the Qinghai Tibetan Plateau under a warming and wetting climate: patterns and mechanism

**DOI:** 10.1093/femsec/fiag093

**Published:** 2026-10-01

**Authors:** Pengyu Li, Baoming Ji, Yaoming Li

**Affiliations:** School of Grassland Science, Beijing Forestry University, Beijing 100083, China; School of Grassland Science, Beijing Forestry University, Beijing 100083, China; School of Grassland Science, Beijing Forestry University, Beijing 100083, China

**Keywords:** climate warming and wetting, alpine meadow, arbuscular mycorrhizal fungi, soil organic carbon, biotic carbon pump

## Abstract

Climate warming accompanied by an overall increase in precipitation has reshaped hydrothermal regimes in many alpine meadows on the Qinghai–Tibetan Plateau, with important implications for soil organic carbon (SOC) dynamics. However, precipitation trajectories are spatially heterogeneous across alpine meadow regions, and the direction and mechanisms underlying SOC responses to warming–wetting conditions remain uncertain. While traditional frameworks emphasize plant litter inputs, emerging evidence highlights the central role of microbial processes in SOC stabilization. Arbuscular mycorrhizal fungi (AMF), which are widely distributed in alpine meadow ecosystems, mediate plant-derived carbon (C) transfer into soils and may regulate SOC formation, turnover, and stabilization. This review synthesizes current knowledge on how warming and increased precipitation influence SOC stocks and dynamics in alpine meadows, with a focus on AMF-mediated mechanisms. We highlight how AMF regulate belowground C inputs, priming effects, and microbial metabolism, thereby affecting SOC accumulation and stabilization. Evidence indicates that warming and altered precipitation jointly modify SOC turnover by influencing plant–AMF C allocation, AMF community composition, microbial C use efficiency, and enzyme activities. These responses are strongly context dependent.We propose an AMF-mediated biotic C pump (BCP) under warming and wetting, emphasizing the role of AMF in promoting the incorporation and stabilization of newly fixed C in soils. This framework advances understanding of C cycling and improves predictions of ecosystem feedbacks to climate change.

## Teaser

This fragmented research landscape creates an urgent need for a systematic synthesis to reconcile these divergent findings and clarify the underlying biological mechanisms.

## Introduction

Global climate change is reshaping the structure and functioning of terrestrial ecosystems at an unprecedented rate and is profoundly altering carbon (C) cycling processes at both regional and global scales (Piao et al. 2010). Among these changes, rising temperature and altered precipitation regimes have emerged as critical environmental drivers governing the dynamics and trajectories of alpine ecosystems. The Tibetan Plateau is widely recognized as a climate-sensitive region and an amplifier of global climate change, exhibiting warming magnitudes and hydrothermal shifts that substantially exceed the global average. Recent climate projections indicate that warming accompanied by increased precipitation is likely to continue across much of the Tibetan Plateau under future Shared Socioeconomic Pathway scenarios (Deng and Ji 2023), supporting the relevance of a warming–wetting framework for this region. Nevertheless, this tendency should not be interpreted as spatially uniform across all alpine meadows, because Tibetan Plateau precipitation also exhibits pronounced regional and seasonal heterogeneity, including a north–south dipole pattern (Ma et al. 2023). Owing to its heightened sensitivity to climatic forcing, the Plateau provides a natural experimental system for elucidating feedbacks between climate change and terrestrial C cycling. Alpine grasslands represent the most extensive and widespread ecosystem type on the Tibetan Plateau. The upper 0–1 m soil layer of these systems stores exceptionally large amounts of soil organic carbon (SOC), accounting for more than 70% of the regional terrestrial C pool (Chen et al. 2022). As such, alpine grasslands play an irreplaceable role in maintaining regional C balance and regulating the global climate system.

In recent decades, climate change on the Tibetan Plateau has generally been characterized by rapid warming accompanied by an overall increase in precipitation, often described as a warming–wetting tendency (Kuang and Jiao 2016, Deng and Ji 2023). However, this regional tendency masks strong spatial and seasonal heterogeneity in precipitation change, as shown by the north–south dipole pattern of Tibetan Plateau precipitation (Ma et al. 2023). Such coupled changes in thermal and moisture regimes not only directly influence plant growth and soil physicochemical properties, but also fundamentally reshape SOC formation, stabilization, and turnover by regulating belowground C inputs and microbial processes. Numerous studies have shown that warming enhances microbial metabolic activity and accelerates organic matter decomposition, thereby stimulating SOC mineralization and CO_2_ release and generating a positive climate–C feedback (Feng et al. 2008, Jia et al. 2017). In contrast, increased precipitation may enhance SOC accumulation and stabilization to some extent by stimulating plant productivity and root biomass, thereby increasing belowground C inputs (Chen et al. 2022). However, the direction and magnitude of SOC responses to climate change vary substantially across regions and hydrothermal contexts, exhibiting pronounced nonlinearity and strong scenario dependence. These discrepancies suggest that SOC responses to warming–wetting are not governed solely by direct climatic forcing, but are largely mediated by biological processes—particularly plant–microbe interactions that regulate C allocation pathways and determine the fate of newly assimilated C.

Within this biological framework, the microbial C pump (MCP) theory proposes that microorganisms contribute to long-term SOC accumulation and stabilization by transforming labile organic C into persistent microbial necromass (Liang et al. 2017, Xiao et al. 2023). While the MCP framework highlights the central role of heterotrophic microbial metabolism and necromass formation in soil C pools, it largely underrepresents the distinctive C acquisition pathways and sequestration strategies of symbiotic microorganisms. In terrestrial ecosystems, belowground symbionts—particularly arbuscular mycorrhizal fungi (AMF)—play a unique and indispensable role in linking plant-derived photosynthetic C inputs to the stabilization of soil C pools. The concept of an “AMF-mediated biotic carbon pump (BCP)” emphasizes the distinctive features of AMF as symbiotic fungi in both C acquisition and C sequestration pathways (Fig. 1). Rather than independently capturing photosynthate, AMF receive recently fixed C from their host plants through symbiotic C exchange and subsequently translocate this plant-derived C into the rhizosphere and hyphosphere via extensive hyphal networks. In addition, AMF promote soil aggregate formation and persistence via hyphal enmeshment and physical encapsulation effects, thereby enhancing the spatial isolation and physical protection of organic C and altering the pathways and efficiency by which C enters stable pools.

**Figure 1:**
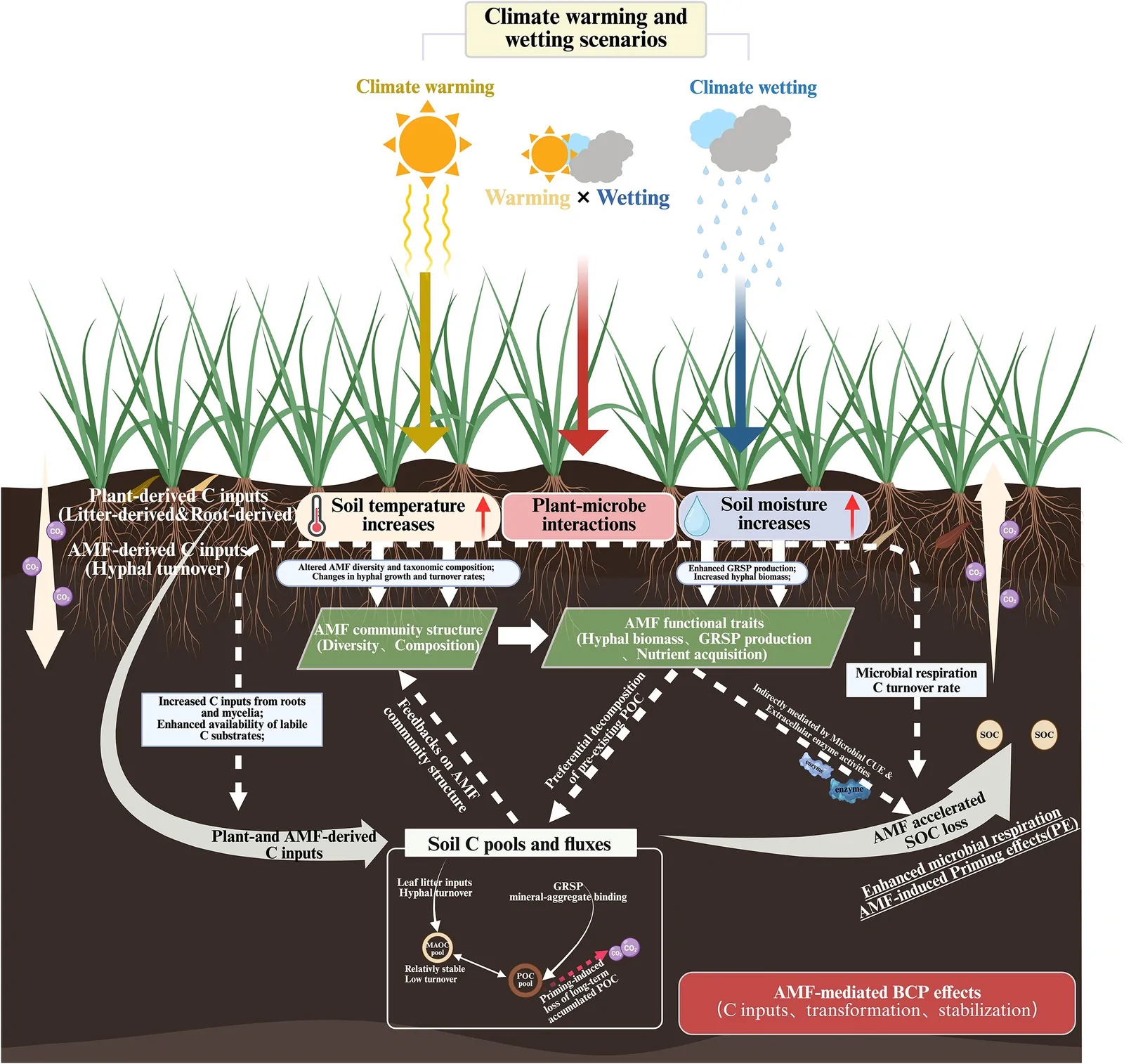
Conceptual framework illustrating the mechanisms by which climate warming and increased precipitation regulate the arbuscular mycorrhizal fungi (AMF)-mediated biotic carbon pump (BCP) in alpine meadows. Climate warming, increased precipitation, and their combined effects modify soil temperature, soil moisture, and plant–microbe interactions, thereby altering AMF community structure (diversity and composition) and AMF functional traits (hyphal biomass, glomalin-related soil protein (GRSP) fractions, and nutrient acquisition). These changes regulate belowground carbon (C) allocation, microbial activity, and soil C transformation, ultimately influencing soil organic carbon (SOC) stabilization through particulate organic carbon (POC), mineral-associated organic carbon (MAOC), aggregate formation, microbial respiration, and AMF-induced priming effects. Plant-derived C inputs (including litter-derived and root-derived C) and AMF-derived C inputs (hyphal turnover) jointly contribute to belowground C pools and fluxes, whereas AMF-mediated biotic processes regulate soil C transformation and stabilization under climate change. Solid arrows indicate processes supported by direct experimental evidence, whereas dashed arrows indicate conceptual or hypothesized pathways that remain incompletely resolved and require further experimental validation. The left, central, and right downward arrows represent the effects of climate warming, their combined effects, and increased precipitation (climate wetting), respectively. Large curved arrows highlight the major directions of belowground C transfer and soil C turnover.

AMF are widely associated with plant roots in alpine grasslands and constitute important conduits for C transfer within plant–soil systems. Plants can allocate a substantial fraction of recently fixed photosynthate to AMF, with experimental estimates reaching up to ∼20% (Jakobsen and Rosendahl 1990). AMF can subsequently transfer part of this plant-derived C into the rhizosphere and hyphosphere through extensive hyphal networks, providing a belowground C input pathway that partly bypasses the conventional litter–decomposition route and may contribute to SOC formation, turnover, and stabilization. Unlike the MCP, which relies predominantly on microbial necromass accumulation, AMF exhibit pronounced functional specificity in C sequestration processes. On the other hand, AMF are associated with the accumulation of glomalin–related soil protein (GRSP)–like, operationally defined soil fractions that may partly originate from hyphal and spore wall residues released during fungal turnover. These AMF-associated soil organic matter (SOM) fractions, together with hyphal enmeshment and other extracellular or wall–derived polymeric materials, can promote soil aggregation and physical protection of organic C, although their biochemical identity and direct origin remain unresolved. Moreover, AMF modulate rhizosphere microbial community composition and metabolic traits, thereby influencing microbial C use efficiency and priming effects, ultimately reshaping SOC turnover rates and stabilization mechanisms (Cheng et al. 2012, Huang et al. 2021, Yang et al. 2024).

Under warming–wetting conditions, AMF responses to increasing temperature and precipitation are highly plastic, with substantial shifts in community structure, functional traits, and plant–mycorrhizal C allocation strategies. These changes influence not only the quantity and quality of belowground C inputs, but also jointly regulate SOC formation and loss by altering microbial metabolic pathways and soil structural properties. Accordingly, AMF should be recognized as a key biological driver distinct from the traditional MCP framework, warranting the explicit formulation of an “AMF-mediated BCP”. This mechanism operates through the symbiotic transfer of recently fixed plant-derived C from host plants to AMF and enhances the efficiency and long-term stability of soil C inputs through hyphal networks, aggregate formation, and rhizosphere regulation, thereby strengthening the buffering capacity of soil C pools against climate change. In particular, the coupling between AMF-mediated processes and heterotrophic microbial dynamics is still not well understood.

Microbial C use efficiency (CUE) and extracellular enzyme activities are key physiological determinants of SOC storage. Climate change may jointly regulate microbial CUE and enzyme activities by altering AMF-mediated C input patterns and rhizosphere environmental conditions, thereby reshaping SOC accumulation potential and turnover pathways. Insufficient attention to these interactions has constrained our understanding of the mechanisms underlying C sink formation in alpine grasslands.

Against this backdrop, this review synthesizes current knowledge on the responses of SOC stocks and turnover processes in alpine grasslands to warming, increased precipitation, and their interactive effects. We place particular emphasis on the ecological mechanisms by which AMF mediate SOC formation and stabilization through regulating belowground C inputs, physical protection, and microbial metabolic processes. Furthermore, we highlight the pivotal role of the AMF-mediated BCP in the formation and persistence of C sinks in alpine grasslands. This synthesis aims to provide a novel theoretical framework and scientific basis for evaluating C sink functions and predicting climate–C feedbacks in alpine regions.

## Effects of a warming–wetting climate on SOC stocks in alpine grasslands of the Tibetan Plateau

### Rationale for investigating SOC dynamics in Tibetan alpine grasslands under a warming–wetting climate

The Tibetan Plateau serves as a critical ecological security barrier for China and much of Asia, and represents one of the most important natural laboratories for studying global change and terrestrial C cycling. Alpine grasslands, the dominant vegetation type on the Plateau, store ∼12 Pg of SOC within the 0–1 m soil profile, making them a central component of the regional C pool (Chen et al. 2022). Over the past five decades, mean annual air temperature on the Tibetan Plateau has increased at a rate of ∼0.46°C per decade, accompanied by an overall rise in annual precipitation ranging from 3.99 to 16.84 mm per decade (Kuang and Jiao 2016). Consistent with this observed tendency, Coupled Model Intercomparison Project Phase 6 (CMIP6)-based projections suggest that warming and wetting will continue over much of the Tibetan Plateau under Shared Socioeconomic Pathway scenarios (Deng and Ji 2023). However, this does not imply uniform wetting across all alpine meadow regions, because precipitation trends on the Plateau are spatially and seasonally structured, including a north–south dipole pattern (Ma et al. 2023).

Climate warming and wetting is not a simple additive effect of increased temperature and precipitation; rather, it fundamentally reshapes the key constraints governing ecosystem C cycling by altering soil hydrothermal coupling, plant physiological regulation pathways, and microbial metabolic environments. Sustained increases in temperature and shifts in water availability jointly influence plant productivity, C allocation patterns, root–hyphal growth, and soil microbial metabolic activity, thereby regulating SOC inputs, transformation pathways, and stabilization processes. Under the combined influence of multiple interacting drivers, changes in SOC stocks often exhibit strong nonlinearity and context dependency, making their direction and magnitude difficult to predict based on any single climatic factor alone.

SOC represents not only the largest organic C reservoir in terrestrial ecosystems, but also a fundamental basis for maintaining soil structural stability, nutrient supply capacity, and ecosystem resilience. Nutrients such as nitrogen (N) and phosphorus (P) released during SOC mineralization directly constrain plant growth and primary productivity, whereas SOC stabilization through aggregate formation and mineral association is strongly dependent on microbial metabolic processes and the accumulation of microbial residues. In alpine ecosystems in particular, low temperatures, frequent freeze–thaw cycles, and pronounced heterogeneity in soil hydrothermal conditions confer strong spatial variability (Lehmann et al. 2020) and temporal lag effects (Koven et al. 2017, Melillo et al. 2017, Liu et al. 2025) in SOC responses to climate change.

Given the vast SOC stocks stored in alpine grasslands, even minor changes in SOC can substantially influence atmospheric CO_2_ concentrations and generate feedbacks to regional and global climate systems. Therefore, developing a systematic understanding of SOC dynamics and their multifactorial regulatory mechanisms in alpine grasslands of the Tibetan Plateau—particularly the non-additive responses arising from coupled changes in temperature and moisture—is essential for accurately assessing the strength of regional C–climate feedbacks.

### Effects of warming on SOC storage in alpine grasslands of the Tibetan Plateau

SOC in alpine grasslands of the Tibetan Plateau is highly sensitive to temperature variation, and climatic warming is widely recognized as a key environmental driver regulating SOC dynamics in this region. A series of field-based warming experiments have demonstrated that an increase in temperature of ∼2°C can lead to a decline of nearly 10% in SOC stocks in alpine grasslands, indicating a potential weakening effect of warming on soil C reservoirs (Davidson and Janssens 2006, Liáng et al. 2022, Tao and Liu 2024, Fan et al. 2026). Mechanistically, elevated temperatures improve soil thermal conditions (Cheng et al. 2017), thereby stimulating microbial metabolic activity and enzyme-mediated reactions. This accelerates organic matter decomposition and soil respiration, ultimately enhancing CO_2_ emissions from soil systems (Feng et al. 2008, Jia et al. 2017). However, the influence of warming on SOC is not uniformly negative. On the one hand, warming can enhance plant productivity by prolonging the growing season and increasing photosynthetic efficiency. This alters above–belowground C allocation patterns, promotes deeper root penetration into soil profiles (Johnson et al. 2006, Leppälammi-Kujansuu et al. 2013, Keuper et al. 2017, Wang et al. 2017, Arndal et al. 2018, Liu et al. 2018, Xiong et al. 2018, Lim et al. 2019), and increases inputs of belowground biomass and root-derived exudates, thereby supplying fresh organic C to soil pools (Cotrufo et al. 2015). On the other hand, microbial metabolic products and necromass constitute critical precursors of mineral-associated organic C (MAOC), whose formation and persistence depend on microbial turnover rates, mineral surface properties, and nutrient availability (Philippot et al. 2024). Under warming conditions, accelerated microbial turnover may simultaneously enhance the mineralization of labile C fractions while facilitating the formation of more stable C pools.

Importantly, warming effects do not occur under constant moisture conditions; instead, their net influence on SOC is strongly contingent upon the prevailing soil moisture regime. Soil moisture regulates plant physiological performance, root growth, and microbial activity, thereby determining whether warming predominantly drives enhanced decomposition or favors increased C inputs and stabilization. Consequently, assessments of warming effects conducted under a single moisture context may underestimate the complexity of SOC responses under realistic climate change scenarios.

At regional and long-term scales, conclusions regarding SOC responses to warming in alpine grasslands of the Tibetan Plateau remain highly divergent. Long-term observations in certain regions have reported increasing SOC contents over the past decade (Chen et al. 2017a). In contrast, other studies suggest that warming substantially enhances SOC mineralization, resulting in sustained losses of soil C stocks (Crowther et al. 2016). Still, meta-analyses and syntheses indicate that the overall effect of warming on SOC may be statistically insignificant (Wang et al. 2022a). Collectively, these contrasting findings highlight the pronounced context dependency of SOC responses to warming in alpine grasslands, governed jointly by spatial scale, experimental duration, vegetation type, and the coupling between soil and microbial processes.

### Regulation of SOC dynamics by precipitation changes in alpine grasslands

Compared with temperature, the effects of precipitation change on SOC are markedly more ecosystem-specific and context-dependent. In arid and semi-arid grasslands, increased precipitation typically alters soil hydrological and biogeochemical processes (Min et al. 2025), alleviating water limitation and enhancing plant photosynthesis and biomass accumulation. This effect is particularly pronounced for belowground biomass production and root exudation, thereby promoting SOC inputs and accumulation (Wang et al. 2020). Under such conditions, enhanced soil moisture can partially offset warming-induced acceleration of SOC decomposition by increasing plant-derived C inputs. In contrast, in alpine meadow ecosystems where baseline soil moisture is relatively high, SOC responses to increased precipitation are far less consistent. Multiple field manipulation experiments and long-term observational studies have shown that even substantial increases in precipitation may result in negligible changes in SOC stocks, or even lead to SOC declines (Chen et al. 2022). This paradox arises because increased soil moisture not only stimulates plant C inputs but can also markedly enhance microbial activity, accelerating organic matter decomposition (Liu et al. 2016) and mineralization processes, thereby increasing soil respiration rates. The resulting C losses may offset, or even exceed, the positive effects of enhanced plant productivity on SOC accumulation.

Therefore, precipitation change does not simply determine whether SOC increases or decreases; rather, it modifies soil hydrothermal conditions and regulates the relative balance between plant C inputs and microbially mediated decomposition under warming. This regulatory mechanism provides critical insights into the nonlinear responses of SOC dynamics under combined warming and wetting scenarios.

### Interactive effects of warming and precipitation on SOC storage

Under a warming and wetting climate regime, changes in temperature and water availability commonly interact to regulate SOC dynamics in alpine grasslands, and their combined effects are rarely a simple sum of single-factor responses. Recent multifactor global change studies have increasingly highlighted the prevalence of non-additive responses (Rongstock et al. 2026), whereby joint effects can be synergistic or antagonistic. This characteristic is particularly pronounced under scenarios involving concurrent changes in heat and moisture. Accumulating evidence indicates that soil moisture strongly modulates warming effects: under relatively moist conditions, warming often enhances plant biomass production (Rongstock et al. 2026), potentially offsetting SOC losses through increased plant-derived C inputs; by contrast, under water-limited conditions, warming tends to intensify evapotranspiration from both plants and soils (Song et al. 2019), thereby accelerating SOC decomposition and C loss (Wang et al. 2022a). Consequently, predictions of future SOC trajectories in alpine grasslands based on single climate drivers alone remain highly uncertain.

When warming induces soil drying, plants may adjust belowground C allocation to maintain water and nutrient acquisition, including altered C investment into mycorrhizal symbioses. If increased precipitation effectively alleviates drought constraints, plant C assimilation and rhizosphere C supply may be enhanced, thereby reshaping AMF-related C allocation pathways—such as extraradical hyphal proliferation, rhizodeposition, and aggregate-associated processes—and their regulation of microbial decomposition and MAOC formation. In this context, the effects of warming × increased precipitation on SOC are more likely to reflect a shift in limiting factors (from water limitation to relative sufficiency) and a reordering of process intensities along the plant input–microbial mineralization–mineral protection continuum, ultimately giving rise to antagonistic or synergistic non-additive responses.

This “non-simple additive” behavior is also supported by warming–precipitation manipulation experiments. For example, in semi-arid temperate grasslands of northern China, warming and increased precipitation exerted strong interactive effects on fine-root production, mortality, and turnover, with response directions contingent upon background moisture conditions. These studies further demonstrated close linkages between root dynamics and soil or ecosystem respiration, indicating that climate drivers influence ecosystem C cycling by reshaping C allocation and belowground inputs (Bai et al. 2010). In alpine meadows, such interactions are likely to be even more complex. On the one hand, increased precipitation may buffer warming-induced soil drying and reduce temperature-stimulated decomposition losses; on the other hand, higher soil moisture may simultaneously enhance microbial activity and substrate diffusion, thereby amplifying mineralization processes and rendering the net SOC response uncertain or even directionally reversed.

Although several studies have examined SOC storage responses in Tibetan Plateau alpine grasslands under warming and altered precipitation, experimental evidence remains limited and conclusions are often inconsistent, with no unified understanding of the underlying mechanisms. Existing research has largely emphasized single-factor effects, while the integrated mechanistic chain linking plant C allocation, root and mycorrhizal inputs, microbial processes, and SOC stabilization under concurrent temperature and moisture changes has rarely been systematically tested—particularly with respect to non-additive responses and their threshold conditions. Meanwhile, soil microorganisms, especially mycorrhizal fungi as key regulators of SOC transformation and stabilization, remain poorly understood in terms of their responses to warming–wetting regimes and their roles in C transfer and retention. Therefore, disentangling mycorrhiza-mediated plant–microbe–soil C pathways within a multifactor warming × precipitation framework represents a critical step toward improving our understanding of SOC dynamics and climate feedbacks in alpine grasslands.

## Roles of AMF in SOC turnover and stabilization

AMF constitute the most widespread mycorrhizal type in terrestrial ecosystems, forming associations with ∼72% of vascular plant species (Brundrett and Tedersoo 2018). They play pivotal roles in plant nutrient acquisition, soil structural stabilization, and the regulation of terrestrial C cycling (Wang and Qiu 2006, Smith and Read 2008, Wang et al. 2022b, Zhang et al. 2022). In alpine meadow ecosystems of the Tibetan Plateau, AMF symbioses are ubiquitous and often characterized by high colonization rates, positioning AMF as a central biological conduit for C transfer and transformation along the plant–soil continuum. Field surveys have reported AMF colonization rates ranging from 20% to 98% in plant roots of alpine grasslands, with *Glomus* species consistently dominating the community (Dong et al. 2021). This widespread and intensive colonization suggests that AMF are deeply involved in the formation, turnover, and long-term stabilization of SOC in these systems (He et al. 2025). Consequently, under the ongoing trend of climate warming and increased precipitation, a mechanistic understanding of AMF-mediated SOC cycling is essential for elucidating C stock dynamics in alpine meadows and for predicting their feedbacks to regional and global climate change.

### AMF-mediated belowground C inputs: the source of the BCP

AMF, belonging to the phylum Glomeromycota, rely entirely on photosynthetically fixed C supplied by their host plants to sustain growth and metabolic activity (Zhu and Michae 2003, Wu et al. 2023). It has been estimated that plants allocate ∼4%–20% of their assimilated C to AMF, resulting in an annual transfer of up to 1.07 Pg C into soils via this symbiotic pathway (Hawkins et al. 2023). This C is not only invested in the expansion and maintenance of extensive hyphal networks but is also continuously delivered to soils in the form of hyphal necromass and a diverse array of fungal metabolites, thereby constituting a persistent input of newly fixed organic C. In natural grassland ecosystems, AMF hyphal densities can reach ∼40–45 m of hyphae per cm³ of soil (Stewart et al. 2026), and AMF-derived inputs are estimated to account for nearly 15% of total SOC stocks (Leake et al. 2004). AMF-associated GRSP-like fractions have often been linked to soil aggregation and SOC persistence (Agnihotri et al. 2021). However, these fractions should be interpreted cautiously because GRSP is an operationally defined, heat-extractable soil fraction rather than a chemically uniform AMF-secreted glycoprotein. Earlier evidence suggests that glomalin-like immunoreactivity is largely associated with AMF hyphae and spores and may enter soil mainly through hyphal turnover and wall degradation rather than direct secretion (Driver et al. 2005). More recent molecular evidence further indicates that the MAb32B11-reactive material may be carbohydrate-rich and derived from AM fungal cell walls, supporting the proposed shift from “glomalin” to “glomalose” (Alptekin et al. 2025). Therefore, GRSP-like fractions can be discussed as AMF-associated indicators linked to aggregation and SOC persistence, but they should not be used as direct evidence for the secretion of a defined protein by living AMF.

### AMF-mediated enhancement of SOC physical stabilization via soil structural regulation

Beyond contributing newly fixed organic C to soils, AMF can indirectly enhance SOC persistence by modifying soil physical structure. Extensive hyphal networks, together with AMF-associated extracellular or wall-derived polymeric materials, can promote aggregate formation and reinforce aggregate stability, thereby increasing the physical protection of SOC and reducing its exposure to microbial decomposition (Wu et al. 2023). Empirical evidence indicates that AMF symbiosis enhances the allocation of photosynthetically derived C to the rhizosphere and surrounding soil, increases the proportion of macroaggregates, and elevates MBC, collectively contributing to greater physical stabilization of SOC (Li et al. 2024).

Inoculation experiments further demonstrate that AMF generally increase plant biomass and SOC stocks (Tao and Liu 2024), suggesting that their contribution extends beyond enhanced belowground C inputs to include structural modification of soil and regulation of microbial habitats, thereby influencing SOC turnover and retention. Compared with C derived from plant litter or roots, AMF-derived C tends to exhibit greater chemical recalcitrance and slower turnover rates (Shi et al. 2023), facilitating its incorporation into more persistent pools such as MAOC or aggregate-protected fractions. Studies along representative grassland transects in China have further shown that AMF community composition and functional traits (Whalen et al. 2024) are closely associated with SOC storage, underscoring the central regulatory role of AMF in grassland C sequestration processes (Zhang et al. 2025).

### AMF regulation of microbial metabolism and priming effects: a double-edged sword for the BCP

AMF exert strong control over rhizosphere microbial metabolism and thereby influence SOC decomposition dynamics (Yuste et al. 2011, Averill et al. 2014). Because AMF extraradical hyphae often have short life spans and rapid turnover rates, hyphal turnover may represent an important pathway by which AMF-associated C enters soil (Staddon et al. 2003a). However, AMF can retract and recycle cytoplasmic materials from senescing hyphae, so dead hyphae may mainly contribute wall-derived residues rather than intact cytoplasmic biomass or a defined suite of extracellular sugars, organic acids, and proteins (Bago et al. 1998, Kokkoris et al. 2020). These AMF-associated residues may alter microbial substrate availability and potentially trigger priming effects in contrasting directions (Cheng et al. 2012, Huang et al. 2021, Yang et al. 2024). However, microbial responses to C inputs cannot be explained solely by substrate supply. Whether greater community complexity—reflected in functional diversity or taxon-specific traits—necessarily enhances decomposition or ecosystem functioning remains debated. In some contexts, increased microbial complexity stimulates decomposition or elevates metabolic activity (Aerts 2006, Strickland et al. 2009), yet no universal, positive relationship exists between diversity and ecosystem functioning (Cox et al. 2001). Importantly, AMF influence extends beyond supplying labile substrates. In association with roots, AMF may redirect C fate by altering mineral-associated processes. For example, root–AMF co-occurrence has been shown to increase dissolved organic C (DOC) concentrations and reactive iron minerals, stimulate litter decomposition and elevate soil CO_2_ efflux, yet simultaneously reduce MAOC. Microscopic evidence indicates that the surfaces of fungal hyphae can be coated with thin layers of iron minerals, suggesting close physical associations between hyphae and soil iron phases. Notably, different AMF genera exhibit contrasting relationships with reactive iron minerals (Li et al. 2023), implying that functional AMF groups may influence SOC stabilization through distinct mineral-mediated pathways.

Accumulating evidence indicates that AMF-mediated priming is strongly context dependent. Under certain conditions, inputs of labile C and rhizosphere stimulation enhance microbial activity and accelerate the mineralization of pre-existing SOC. Under other scenarios, AMF may promote C persistence by restructuring microbial communities, increasing microbial C use efficiency, intensifying substrate competition, or facilitating the mobilization of mineral-bound nutrients while promoting the formation of reactive mineral phases. In such cases, AMF activity may manifest as negative priming (Carrillo et al. 2016). Moreover, temporal fluctuations in soil moisture, temperature, nutrient availability and organic C inputs can further induce nonlinear responses in SOC decomposition (Lehmann et al. 2020). AMF therefore cannot be viewed as uniformly accelerating or suppressing SOC turnover. Rather, they modulate underground C fluxes by reorganizing the linkage among substrate supply, microbial metabolism and mineral association/protection under varying environmental contexts. Through this reconfiguration, AMF shape microbial metabolic strategies and C allocation patterns, ultimately influencing net SOC accumulation and long-term stability

## Effects of warming, increased precipitation, and their interactions on AMF-mediated BCP processes

Under ongoing global change, many alpine meadow regions on the Qinghai–Tibetan Plateau are experiencing rapid warming accompanied by increased or altered precipitation, a pattern often discussed within a warming–wetting framework. However, because precipitation changes are not spatially uniform across the Plateau, the AMF-mediated BCP should be considered within region-specific hydroclimatic contexts rather than under a single homogeneous moisture trajectory (Ma et al. 2023). In natural settings, temperature and water availability rarely shift in isolation; instead, their co-variation is likely to reshape belowground C inputs by altering C exchange between plants and AMF. However, most existing studies have examined warming and increased precipitation separately, or have focused primarily on plant productivity and total SOC stocks. As a result, substantial uncertainty remains regarding how AMF-mediated BCP processes respond to these interacting climatic drivers (Bennett and Classen 2020).

### Warming drives BCP inputs by reshaping plant–AMF C allocation and functional strategies

Under relatively favorable moisture conditions, warming generally enhances AMF-mediated C inputs by stimulating plant photosynthesis and increasing belowground C allocation. Numerous studies have shown that elevated temperature can increase AMF root colonization rates and extraradical hyphal biomass, while promoting C transfer from host plants to their fungal partners (Heinemeyer and Fitter 2004, Gavito et al. 2005). This enhanced allocation not only increases the flux of newly assimilated plant C into soil—by as much as 10%–100% in some systems (Staddon et al. 2004, Hawkes et al. 2008, Liu et al. 2018, Zhang et al. 2020b)—but also facilitates sustained organic C inputs through hyphal proliferation and the accumulation of fungal residues. Together, these processes strengthen the input component of the BCP.

Importantly, warming is frequently accompanied by marked shifts in AMF taxonomic composition and functional traits, with potential consequences for the efficiency and stability of the BCP (Hawkes et al. 2008, Chaudhary et al. 2022). Such restructuring may indicate a transition from relatively conservative (K-strategist) taxa toward more opportunistic (r-strategist) taxa. Empirical evidence suggests that warming favors taxa characterized by rapid hyphal growth and high C acquisition efficiency—such as members of *Paraglomeraceae* and *Diversisporaceae*—while suppressing *Glomeraceae*, which typically exhibit lower C demand, limited extraradical hyphal extension, and high levels of intraradical colonization (Qiu et al. 2021, Xu et al. 2021). These community shifts are typically associated with faster hyphal turnover and more rapid utilization of newly fixed C—traits consistent with the “high growth–high consumption–low persistence” profile of r-strategist fungi. From the perspective of long-term SOC stabilization, however, this functional transition may have contrasting implications. On the one hand, the expansion of r-strategist AMF can enhance short-term C flux from plants to soil. On the other hand,

Glomeraceae have often been associated with higher accumulation of GRSP-like fractions and soil aggregate formation (Rillig et al. 2002, Poeplau et al. 2016); a decline in their relative abundance may weaken the physical protection and mineral association of SOC, thereby reducing the structural stability of soil C pools. Long-term warming experiments in alpine grasslands on the Qinghai–Tibetan Plateau have indeed reported reductions in soil aggregate fractions accompanied by declines in SOC stability (Guan et al. 2018), lending empirical support to this mechanism.

In contrast, under moisture limitation or imbalanced hydrothermal conditions, the positive effects of warming on the AMF-mediated BCP are often weakened or even reversed. In arid and semi-arid systems, warming can reduce plant C allocation to AMF, leading to lower hyphal density and simplified mycelial networks, thereby diminishing AMF-driven belowground C inputs (Rillig et al. 2002, Staddon et al. 2004). Moreover, differential responses among plant functional types further increase this variability. Long-term prairie experiments in North America, for instance, have shown that warming markedly increases hyphal density in AMF associated with C_4_ species, while exerting limited effects on colonization rates in C_3_ plants (Rudgers et al. 2014). Taken together, warming-induced shifts in AMF-mediated C fluxes are strongly modulated by water availability and by changes in AMF functional composition, ultimately influencing SOC turnover pathways and long-term stability.

### Increased precipitation modulates the BCP by reshaping AMF communities and plant C allocation strategies

Compared with temperature effects, considerably less attention has been paid to how altered precipitation regulates AMF-mediated C inputs (Bennett and Classen 2020). Nevertheless, emerging evidence indicates that sustained shifts in soil moisture can restructure microbial community composition and metabolic strategies, thereby modifying AMF colonization intensity and the dynamics of C exchange between fungi and their host plants.

Long-term alterations in precipitation regimes have been shown to markedly influence microbial community composition (Min et al. 2025) and decomposition processes. For instance, a 14-year irrigation experiment enhanced microbial decomposition of N-rich substrates such as chitin and proteins, ultimately reducing soil organic matter stocks (Wang et al. 2023). These findings suggest that increased moisture may accelerate microbially mediated C turnover, thereby indirectly constraining mycorrhiza-associated C sequestration. Conversely, prolonged drought has been associated with higher fungal-to-bacterial ratios, improved microbial C use efficiency, and reduced respiratory C losses (Zeglin et al. 2013), implying a functional shift toward greater C retention under water limitation. Within a moderate range, enhanced precipitation can alleviate warming-induced soil desiccation, thereby improving the physiological performance of both AMF and their host plants. Because microbial growth and metabolism typically respond more rapidly to changes in moisture than plants do (Prosser et al. 2007), moderate water inputs often increase AMF colonization rates and extraradical hyphal biomass, facilitating greater allocation of photosynthate belowground and enhancing SOC inputs via the mycorrhizal pathway (Gao et al. 2016, Chen et al. 2017b). Under such conditions, increased moisture may partially offset warming-driven drought stress, enabling plants to sustain or even intensify C allocation to AMF (Chen et al. 2017b, Deveautour et al. 2018), ultimately strengthening the BCP.

However, the direction and magnitude of AMF responses to altered precipitation are far from uniform and strongly depend on the intensity, duration, and legacy of hydrological change. Some studies report that water addition reduces the relative abundance of *Claroideoglomeraceae* while favoring *Glomeraceae* (Chen et al. 2017b), whereas others detect no significant shifts in community composition (Wei et al. 2021). Similarly, soils with a history of prolonged drought often exhibit sustained pulses of CO_2_ release upon rewetting, with persistent differences in respiratory responses (Liu et al. 2022), indicating that precipitation regimes can leave long-lasting “legacy effects” on microbial C metabolism. Such variability likely arises from the combined influence of warming and increased precipitation on plant photosynthetic capacity, C allocation strategies (Martinez Garcia et al. 2012), and community functional composition (Li et al. 2015), which together may indirectly regulate AMF biomass partitioning and community assembly. In ecosystems such as alpine meadows, where thermal and hydrological conditions are highly heterogeneous, extreme precipitation events may further amplify uncertainties in C cycling under warming scenarios. On the one hand, short-term moisture pulses can stimulate microbial and AMF activity, promoting belowground C inputs from plants. On the other hand, excessive precipitation may accelerate SOC mineralization, enhance dissolved organic C leaching, and increase CO_2_ efflux, thereby weakening the net C sequestration capacity of the AMF-mediated BCP (Wang et al. 2023). Consequently, AMF responses to increased precipitation are likely governed by interactive effects among warming, plant C allocation strategies, and soil physicochemical conditions, resulting in pronounced context dependency.

### Synergistic and antagonistic effects of warming–precipitation interactions on the AMF-mediated BCP

In natural ecosystems, rising temperatures are frequently accompanied by shifts in precipitation regimes, and their combined effects on the AMF-mediated BCP cannot be interpreted as a simple sum of single-driver responses. Rather, temperature and moisture jointly reshape plant–AMF C allocation patterns and may generate synergistic or antagonistic responses. When warming coincides with increased water availability, additional precipitation can offset warming-induced evapotranspiration and soil desiccation, thereby maintaining a more stable soil moisture regime. This buffering effect alleviates water stress on AMF hyphal growth and functional maintenance (Liu et al. 2017, Shaw and Cleveland 2020, Wang et al. 2023) and may enhance host C allocation to the fungal partner. Under such conditions, warming-stimulated photosynthesis and belowground C inputs can be more consistently channelled into the AMF symbiotic pathway in the presence of adequate moisture. This promotes extraradical hyphal proliferation and the accumulation of hyphal residues, while facilitating the transformation of newly fixed C into MAOC pools (Zhou et al. 2022, Ao et al. 2025, He et al. 2025). This coordinated sequence—moisture buffering, enhanced C input, and improved stabilization efficiency—supports the sustained functioning of the AMF-driven BCP. Moreover, warming and wetting may restructure AMF communities in ways that alter BCP efficiency and stability. Functional groups with greater C acquisition and utilization capacities may increase in relative abundance under elevated temperatures, whereas taxa with lower C demand and limited hyphal expansion may decline (Qiu et al. 2021, Xu et al. 2021). If warming concurrently reduces GRSP-associated aggregate formation, the physical protection of SOC may weaken, ultimately compromising long-term C stability (Rillig et al. 2002, Poeplau et al. 2016).

However, when warming and increased precipitation are mismatched in magnitude or timing, their interaction may shift from synergistic to antagonistic. Excessive water inputs can reduce soil aeration or diminish plant reliance on mycorrhizal symbiosis, thereby suppressing AMF colonization rates and hyphal network development, potentially offsetting the positive effects of warming (Wang et al. 2023). In addition, warming under moist conditions often accelerates microbial respiration and organic matter decomposition. If AMF-mediated C inputs fail to compensate for these enhanced losses, the soil C balance may shift from net accumulation to net release (Qiu et al. 2023). Such nonlinear responses are particularly pronounced in nutrient-limited alpine meadow ecosystems, where shifts in plant functional composition and intensified microbial competition can further modify C cycling trajectories.

Overall, the impact of concurrent warming and precipitation change on the AMF-mediated BCP fundamentally depends on the degree of hydrothermal synchrony and its regulation of plant–AMF C exchange strategies. Under a warming–wetting regime, synergistic or imbalanced temperature–moisture interactions alter plant functional group composition, AMF community structure, and soil physicochemical conditions, collectively determining whether AMF enhance or constrain SOC input pathways and stabilization mechanisms.

## Effects of warming and increased precipitation on AMF-mediated BCP processes

### Regulation of AMF-mediated SOC turnover and BCP efficiency under warming

Numerous studies have shown that rising temperature often intensifies AMF-induced priming of SOC, thereby accelerating SOC mineralization. However, when interpreted from the perspective of the BCP, this process should not be viewed solely as a unidirectional C loss pathway. Rather, it represents a critical step through which AMF facilitate the transfer of photosynthetically fixed C from plants into soil and regulate its subsequent redistribution within the soil matrix. Experimental evidence indicates that warming significantly increases the respiration rate and turnover frequency of AMF extraradical hyphae, thereby elevating fungal C demand. This enhanced demand can stimulate stronger positive priming effects and accelerate the decomposition of pre-existing SOC (Fitter et al. 2004, Heinemeyer et al. 2006), potentially resulting in net soil C losses (Hawkes et al. 2008, Qiu et al. 2021). Importantly, such outcomes more likely reflect a temporary disequilibrium among different components of the BCP rather than a weakening of AMF functional capacity per se.

Shifts in AMF community composition under warming are considered a key biological mechanism regulating BCP performance. Several studies have reported that warming tends to decrease the relative abundance of Glomus, while significantly increasing *Claroideoglomus* (Qiu et al. 2021). Because *Glomus* species have often been associated with greater accumulation of GRSP-like fractions, they may contribute to soil aggregation and the physical protection of SOC. In contrast, *Claroideoglomus* typically develops more extensive extraradical hyphal networks and is more efficient in exploiting fresh C substrates, which may enhance priming and SOC turnover. Consequently, warming-induced functional shifts within AMF communities may redirect the BCP from a “stabilization-oriented” mode toward a more “turnover-oriented” state, thereby reducing long-term SOC sequestration efficiency.

Functional divergence among AMF taxa in regulating priming and BCP processes has also been demonstrated experimentally. Cheng et al. (2012) showed that under elevated CO_2_ conditions, soils inoculated with *Acaulospora morrowiae* did not exhibit significant priming, whereas inoculation with *Gigaspora margarita* markedly enhanced SOC decomposition (Cheng et al. 2012). These findings underscore that phylogenetic divergence among AMF taxa profoundly influences functional trade-offs among C input, priming intensity, and C stabilization, ultimately determining the efficiency of the BCP.

Warming may further influence AMF-mediated C regulation by altering fungal morphological traits. Studies have reported that elevated temperature often reduces the proportion of vesicles and spores formed within roots, while simultaneously promoting the expansion of extraradical hyphae (Hawkes et al. 2008, Cotton 2018). This structural transition from a “storage-oriented” to an “exploration-oriented” strategy may facilitate C translocation over larger spatial scales and enhance the activation and reprocessing of soil C pools, thereby strengthening the C transformation component of the BCP (Olsson and Johnson 2005). Nevertheless, some studies have reported no significant warming-induced changes in AMF-driven priming (Shahzad et al. 2015), indicating that temperature effects are strongly context-dependent and jointly regulated by soil moisture, nutrient availability, and the structure of coexisting microbial communities (Staddon et al. 2003b, Kuzyakov 2010, Wei et al. 2019).

### Effects of increased precipitation on AMF-mediated SOC turnover and the BCP

Compared with warming effects, the mechanisms by which increased precipitation regulates AMF-mediated SOC decomposition and BCP processes remain insufficiently resolved. Nevertheless, accumulating evidence suggests that enhanced water availability can strengthen AMF-driven SOC turnover through multiple, interacting pathways. Improved soil moisture enhances microbial and fungal metabolic activity (Zak et al. 1999, Tucker et al. 2013, Liu et al. 2016) and increases substrate diffusion efficiency (Siebielec et al. 2020), thereby intensifying competition and interaction between AMF and heterotrophic microorganisms for labile C resources. Under wetter conditions, freshly assimilated photosynthates delivered to soil via AMF extraradical hyphae are more readily utilized by microbial communities, stimulating respiration and organic C mineralization and accelerating SOC reprocessing. Within the MCP framework, this reflects a rapid transfer of plant-derived C into microbial pathways following its entry into soil. Increased precipitation may also reduce aggregate stability and weaken the spatial protection of organic C within soil structure (Chen et al. 2019), exposing previously protected SOC fractions and lowering their resistance to decomposition (Dimoyiannis 2009). As aggregates loosen and diffusion constraints diminish, the synergistic exploitation of SOC by AMF–microbial consortia is likely enhanced, further accelerating C turnover rates.

In addition, increased moisture may indirectly regulate the BCP by reshaping C allocation strategies within the plant–AMF symbiosis. Under conditions of reduced resource limitation, plant dependence on AMF for nutrient acquisition may decline, while the C cost of maintaining fungal hyphal growth and metabolism increases. Consequently, AMF may shift functionally from primarily facilitating nutrient acquisition to acting as active agents of soil C transformation. This functional reorientation could potentially reduce long-term SOC stabilization, while simultaneously accelerating the conversion of newly derived organic C into mineral-associated forms or microbial necromass. Overall, increased precipitation may reinforce AMF-mediated SOC turnover through enhanced microbial activity, weakened physical protection of C, and altered plant–AMF C allocation patterns. Its net effect on BCP efficiency is therefore likely to be strongly context dependent across ecological settings.

## Key drivers and mechanisms regulating AMF-mediated BCP effects and SOC storage

Under ongoing warming and increased precipitation, the influence of AMF on SOC storage in alpine meadows cannot be attributed to a single process. Rather, AMF reshape belowground C inputs, microbial C allocation strategies, and organic matter decomposition pathways, thereby collectively regulating SOC formation, transformation, and stabilization. Increasing evidence identifies microbial CUE and extracellular enzyme systems as pivotal process-level variables linking climate change, AMF symbiosis, and SOC dynamics. These two components constitute the central regulatory axes of the AMF-mediated BCP (Yu et al. 2025).

### Microbial CUE as a core regulator of BCP strength

Microbial CUE reflects the proportion of assimilated organic C that is incorporated into biomass rather than lost via respiration, thereby determining whether C is retained in soils or returned to the atmosphere (Luo et al. 2024, Yu et al. 2025, Zhou et al. 2026). Empirical evidence across ecosystems shows that both the direction and magnitude of SOC responses to climate change closely track shifts in microbial CUE (Domeignoz Horta et al. 2020), underscoring its predictive value for SOC dynamics (Tao et al. 2023). Within AMF-associated systems, host plants continuously allocate recently fixed photosynthate to the rhizosphere via root exudation and hyphal transport. This targeted and sustained C supply can shift microbial allocation strategies toward biomass production. Compared with non-mycorrhizal systems, AMF-dominated rhizospheres often favor greater incorporation of C into microbial biomass, thereby enhancing the contribution of microbial necromass to SOC formation. This mechanism forms a fundamental basis of the BCP.

Warming commonly accelerates microbial metabolism and respiration, thereby reducing CUE and potentially limiting SOC accumulation (Sinsabaugh et al. 2016, Domeignoz Horta et al. 2020). However, responses of CUE to elevated temperature remain inconsistent, with some studies reporting weak or negligible effects (Zhang et al. 2024). Such variability suggests that temperature effects are modulated by C input rates, nutrient availability, and intrinsic microbial allocation strategies. In AMF-associated systems, preferential C allocation within the plant–fungal partnership may partially offset warming-induced declines in CUE. Moreover, short-term or moderate warming may be insufficient to disrupt established metabolic trade-offs, whereas prolonged or extreme warming scenarios are more likely to reveal pronounced temperature sensitivities. These considerations underscore the need for long-term field manipulations.

Compared with temperature, moisture effects on microbial CUE are more complex yet often directionally consistent. Drought typically reduces soil water availability (Manzoni et al. 2012, Stocker 2013) and suppresses microbial CUE (Sinsabaugh et al. 2013), largely because water stress elevates the metabolic costs associated with osmotic regulation and stress defense, increasing respiratory C losses. Additionally, reduced substrate diffusion under dry conditions further constrains biomass production. Nevertheless, under prolonged drought, microbial communities may adjust compositionally and functionally to maintain relatively stable CUE levels (Fuchslueger et al. 2019, Domeignoz Horta et al. 2020). In contrast, evidence regarding enhanced moisture remains limited, particularly from long-term field irrigation experiments. Under a warming–wetting trajectory, moisture effects on CUE are likely to be highly context-dependent, emerging from interactions among C inputs, nutrient availability, and AMF community characteristics.

### Extracellular enzyme systems: an indirect pathway linking AMF to SOC turnover

Extracellular enzyme activity is a pivotal control on SOC decomposition and stabilization. Although AMF are closely associated with changes in extracellular enzyme activities, current evidence indicates that they do not themselves produce substantial amounts of extracellular enzymes involved in the degradation of complex soil organic matter. Instead, AMF influence extracellular enzyme activities indirectly by modifying host plant C allocation, rhizosphere conditions, and the composition and metabolic activity of associated microbial communities (Duan et al. 2024). Consequently, the effects of AMF on extracellular enzyme-mediated SOC turnover should be regarded primarily as indirect rather than direct. Micro-organisms and plants dynamically regulate the synthesis and secretion of extracellular enzymes in response to nutrient demand and environmental stress, thereby reshaping decomposition pathways and altering the efficiency of organic matter turnover (Sardans and Peñuelas 2005, Sardans et al. 2006, Sinsabaugh and Shah 2012, Burns et al. 2013, Fanin et al. 2016, Zhang et al. 2020a). During SOC turnover, carbohydrate-hydrolyzing enzymes such as cellulases and β-1,4-glucosidase (β-glucosidase, βG) directly regulate substrate availability and are therefore central determinants of SOC mineralization (Jian et al. 2016). Among these, βG is widely used as a sensitive enzymatic indicator of SOC turnover because it catalyzes the degradation of cellulose and soluble polysaccharides, thereby controlling the release of labile C substrates (Debosz et al. 1999, Sardans et al. 2008). However, SOC decomposition and stabilization are not governed by C-hydrolyzing enzymes alone but are tightly coupled with N and P cycling. Enzymes such as proteases, ureases, and phosphatases regulate nutrient regeneration, thereby influencing plant productivity and root-derived C inputs, which ultimately shape long-term SOC accumulation (Sardans and Peñuelas 2005, Vranova et al. 2013). SOC dynamics should therefore be viewed as the emergent outcome of coordinated C–N–P interactions rather than as a C-driven process in isolation.

Temperature and soil moisture are primary environmental regulators of the spatial and temporal dynamics of soil enzyme activity and thus directly influence SOC transformation. Meta-analyses conducted under global change scenarios indicate that warming generally enhances the activity of N-acquiring enzymes, such as N-acetylglucosaminidase (Xiao et al. 2018), and simultaneously stimulates the expression of multiple hydrolytic enzymes, accelerating SOC decomposition and mineralization. In contrast, moisture-mediated regulation of soil enzyme activity is more complex. By altering substrate diffusion, oxygen availability, and microbial metabolic conditions, changes in water availability often induce nonlinear enzymatic responses (Sardans et al. 2008, Zuo et al. 2018). Meta-analyses across ecosystems further demonstrate that enzyme functional groups differ markedly in their sensitivity to precipitation shifts (Min et al. 2025). Increased precipitation tends to stimulate oxidative enzyme activity while exerting limited influence on hydrolytic enzymes; conversely, reduced precipitation significantly suppresses oxidative enzymes but may enhance hydrolytic enzyme activity (Ren et al. 2017). These contrasting patterns reflect the differential dependence of enzyme classes on oxygen availability and substrate accessibility during C decomposition.

Long-term field warming experiments further highlight the interactive regulation between temperature and moisture. Under water-limited conditions, the stimulatory effect of warming on enzyme activity is markedly attenuated, indicating that moisture availability can constrain microbial responses to elevated temperature. Although increased precipitation often enhances microbial activity and enzymatic processes (Qadeer et al. 2022), excessive moisture or water saturation may restrict oxygen diffusion, thereby suppressing aerobic decomposition and reducing SOC breakdown rates (Zuccarini et al. 2020). In peatlands and other chronically wet ecosystems, short-term drought can paradoxically stimulate oxidative enzyme activity by increasing soil oxygen availability. Importantly, moisture-driven changes in soil enzymatic processes are not always reflected in total enzyme activity. Under arid and semi-arid conditions, the Km values (substrate affinity constants) of β-glucosidase and acid phosphatase decline significantly with reduced precipitation, suggesting that microorganisms may alter isoenzyme composition to enhance substrate affinity under water limitation (Chen et al. 2025). Collectively, these findings indicate that microbial communities can adjust enzyme type, quantity, and kinetic properties to buffer environmental variability. Overall, temperature and moisture do not act independently but interactively regulate soil enzymatic processes, jointly defining the environmental constraints that shape SOC decomposition trajectories.

### Context-dependent switching between C sequestration and decomposition-promoting functions of AMF under warm–wet scenarios

Although AMF are widely recognized for enhancing belowground C inputs and promoting SOC stabilization, accumulating evidence indicates that their influence on SOC turnover is not unidirectional but strongly context-dependent (Frey et al. 2013, Cotrufo et al. 2019, Sokol et al. 2019). Depending on environmental conditions, AMF may either strengthen the BCP by facilitating C incorporation into stable pools or, conversely, stimulate the decomposition of pre-existing SOC through priming effects (Cheng et al. 2013, Liu et al. 2020). Importantly, this functional shift is not stochastic; rather, it reflects coordinated changes in soil moisture, nutrient availability, and plant–microbial C allocation strategies under warm–wet climatic conditions (Frey et al. 2013, Terrer et al. 2016).

Under nutrient-limited conditions—particularly when P availability is low—plants rely more heavily on mycorrhizal symbiosis and allocate a greater fraction of photosynthate to AMF in exchange for improved nutrient acquisition (Smith and Smith 2011, Jiang et al. 2017). In such contexts, AMF function predominantly as C sink facilitators. Extraradical hyphae and their residues contribute continuous inputs of newly fixed C to the soil, while the accumulation of AMF-associated GRSP-like fractions and enhanced aggregate formation may strengthen the physical protection of SOC. These processes promote the conversion of microbial residues into MAOC, thereby increasing SOC persistence and residence time (Rillig et al. 2015, Cotrufo et al. 2019). At this stage, AMF-mediated C fluxes are characterized primarily by input and stabilization, with the BCP playing a dominant role (Sokol et al. 2019).

In contrast, when warm–wet conditions substantially increase soil moisture and N and P availability, plant dependence on mycorrhizal nutrient acquisition may decline, while the C cost of maintaining fungal biomass increases (Frey et al. 2013, Fernandez and Kennedy 2016). Under such circumstances, AMF may accelerate hyphal turnover and release labile C substrates, thereby stimulating rhizosphere microbial activity and extracellular enzyme production. This cascade can trigger positive priming effects and enhance the decomposition of existing SOC (Cheng et al. 2013, Liang et al. 2017). Increased soil moisture may further destabilize soil aggregates, weakening physical protection mechanisms and rendering SOC more accessible to microbial degradation (Six et al. 2004, Liu et al. 2020). During this phase, the functional emphasis of AMF shifts from C retention to C reprocessing, with decomposition-promoting effects prevailing. Thus, AMF-mediated SOC dynamics should not be viewed in binary terms of stimulation or suppression; rather, they follow a continuum from nutrient-limited C stabilization to nutrient-replete decomposition enhancement (Fernandez and Kennedy 2016, Terrer et al. 2016). Under ongoing warm–wet trends, shifts in moisture regimes, N/P availability, and plant C allocation strategies together define critical environmental thresholds governing this functional transition (Frey et al. 2013, Jiang et al. 2017, Liu et al. 2020). This scenario-switching framework underscores the conditional nature of AMF-mediated BCP processes, with net effects on alpine meadow SOC stocks determined by the relative reordering of C inputs, microbial metabolism, and stabilization pathways (Six et al. 2004, Sokol et al. 2019).

From a predictive standpoint, overlooking this context dependence may lead to systematic biases when estimating the C sink potential of alpine meadows under warm–wet climate trajectories (Terrer et al. 2016). Future research should adopt multifactorial experimental designs integrating nutrient manipulations, moisture regulation, and functional trait analyses of AMF taxa to identify the thresholds and temporal scales governing the transition from C sink facilitation to decomposition promotion. Such efforts are essential for refining mechanistic predictions of terrestrial C cycle feedbacks.

### Integrative role of AMF in the BCP

Beyond abiotic controls, plant functional types and their associated symbionts—particularly AMF—exert an additional layer of regulation over SOC turnover by reshaping rhizosphere C inputs and extracellular enzyme profiles. Evidence indicates that rhizospheres of AMF-associated vegetation often exhibit elevated activities of C-degrading enzymes coupled with comparatively lower N-acquiring enzyme activities, resulting in a higher C: N ratio of organic matter decomposition (Yin et al. 2021). This enzymatic signature suggests that AMF may enhance C substrate hydrolysis while modulating nutrient limitation regimes, thereby favoring the formation of microbial residue-derived C. Through these coordinated processes, AMF-dominated systems may establish a distinct SOC balance between decomposition and stabilization compared with non-mycorrhizal systems.

In the context of concurrent warming and increased moisture availability, the AMF-mediated BCP emerges from the coupling of microbial CUE, extracellular enzyme systems, environmental constraints, and plant–fungal interactions. Examining nutrient-acquiring enzyme dynamics and their responses to warm–wet scenarios offers mechanistic insight into SOC decomposition pathways and stabilization processes in alpine meadows. Such process-level understanding is essential for improving assessments of terrestrial C sink capacity under climate change (Qi et al. 2016) and for refining predictions of ecosystem C cycling and climate feedbacks (Kowalchuk 2012, Parihar et al. 2020).

## Conclusions

Under ongoing warming and spatially heterogeneous precipitation change on the Qinghai–Tibetan Plateau, the formation, stabilization, and turnover of SOC in alpine meadows are undergoing systemic reorganization. The magnitude and even direction of SOC responses appear highly context-dependent and scale-sensitive. Synthesizing current evidence, AMF emerge as pivotal regulators linking plant photosynthetic C inputs, rhizosphere microbial metabolism, and soil C stabilization processes. On the one hand, AMF enhance belowground C allocation, accelerate hyphal biomass turnover, and are associated with the accumulation of GRSP-like fractions, thereby sustaining the influx of newly fixed C into soil pools. On the other hand, by improving soil aggregate structure, reinforcing physical protection, and modulating rhizosphere microbial CUE and extracellular enzyme systems, AMF actively mediate the transfer of organic C from labile pools to more stable fractions, particularly MAOC. Building on this framework, we propose the concept of an “AMF-mediated BCP”, emphasizing the coordinated, multi-pathway mechanisms through which AMF facilitate long-term subsurface C sequestration. This perspective provides a microbial-process-based lens for interpreting the complex responses of alpine meadow SOC to climate change.

### Future outlook

To date, most investigations of AMF–SOC interactions remain largely correlational, limiting robust inference of causal mechanisms. Under concurrent warming and increased precipitation, AMF may simultaneously enhance SOC stabilization and stimulate priming effects; however, their relative contributions across temporal scales, C input pathways (root-derived inputs, hyphal turnover, GRSP production), and C pool destinations (aggregate occlusion versus MAOC formation) remain unresolved. Disentangling these pathways requires partitionable and comparable quantitative evidence. Moreover, key parameters—including hyphal turnover rates, the origin and persistence of GRSP, microbial necromass formation efficiency, and measurements of CUE and extracellular enzyme kinetics—lack methodological standardization. This inconsistency hampers cross-study synthesis and mechanistic validation.

Future research should prioritize the construction of a quantifiable mechanistic chain linking plant C fixation to stabilized SOC pools under long-term in situ warming–moisture manipulation experiments. Continuous stable isotope tracing (e.g. ^13^C labeling), integrated with high-throughput sequencing and C fractionation approaches (aggregate fractionation and POC versus MAOC separation), will allow direct quantification of C flux through AMF-associated pathways into distinct SOC reservoirs. Such approaches are essential for determining the proportional contribution of AMF-associated C inputs to MAOC formation and persistence. Concurrent standardized measurements of AMF functional traits—such as hyphal length density and turnover rates—together with the origin, biochemical identity, persistence, and accumulation of AMF-associated GRSP-like fractions are needed to support mechanistic comparisons and model parameterization. These measurements should be combined with microbial metabolic attributes, including CUE and extracellular enzyme activity spectra, to clarify how AMF-associated inputs are transformed, mineralized, or stabilized under changing hydroclimatic conditions. Because future precipitation trajectories across alpine meadow regions of the Qinghai–Tibetan Plateau are spatially heterogeneous, future studies should further combine alpine meadow distribution maps with downscaled CMIP6 precipitation projections to quantify the proportions of meadow areas likely to become wetter or drier under future warming. This spatially explicit approach would help evaluate whether AMF-mediated SOC response frameworks developed under warming–wetting conditions are transferable to alpine meadow regions experiencing drying or seasonally imbalanced moisture availability. Ultimately, AMF processes must move beyond conceptual synthesis toward formal representation within terrestrial ecosystem and global change models. Incorporating AMF functional traits, microbially mediated processes, GRSP-like fraction uncertainty, and region-specific hydroclimatic trajectories into soil C submodules will improve predictions of SOC dynamics and climate feedbacks in alpine meadows. Through the integration of experiments, field observations, spatial climate projections, and modeling across scales, we can more fully resolve the ecological significance of the AMF-mediated BCP under ongoing warming and spatially heterogeneous moisture change.
